# Supplementary material for: Community-tailored One Health educational intervention to enhance knowledge and practices for zoonotic disease prevention in rural Thailand: A protocol for a prospective cluster randomised controlled Trial in Chanthaburi, Thailand (Saan Suk trial)
Source: PLoS One. 2026 Sep 15;21(9):e0354989. doi: 10.1371/journal.pone.0354989 (PMC13577416; doi:10.1371/journal.pone.0354989)
Supplement: Supplementary file 1 [file pone.0354989.s002.pdf]

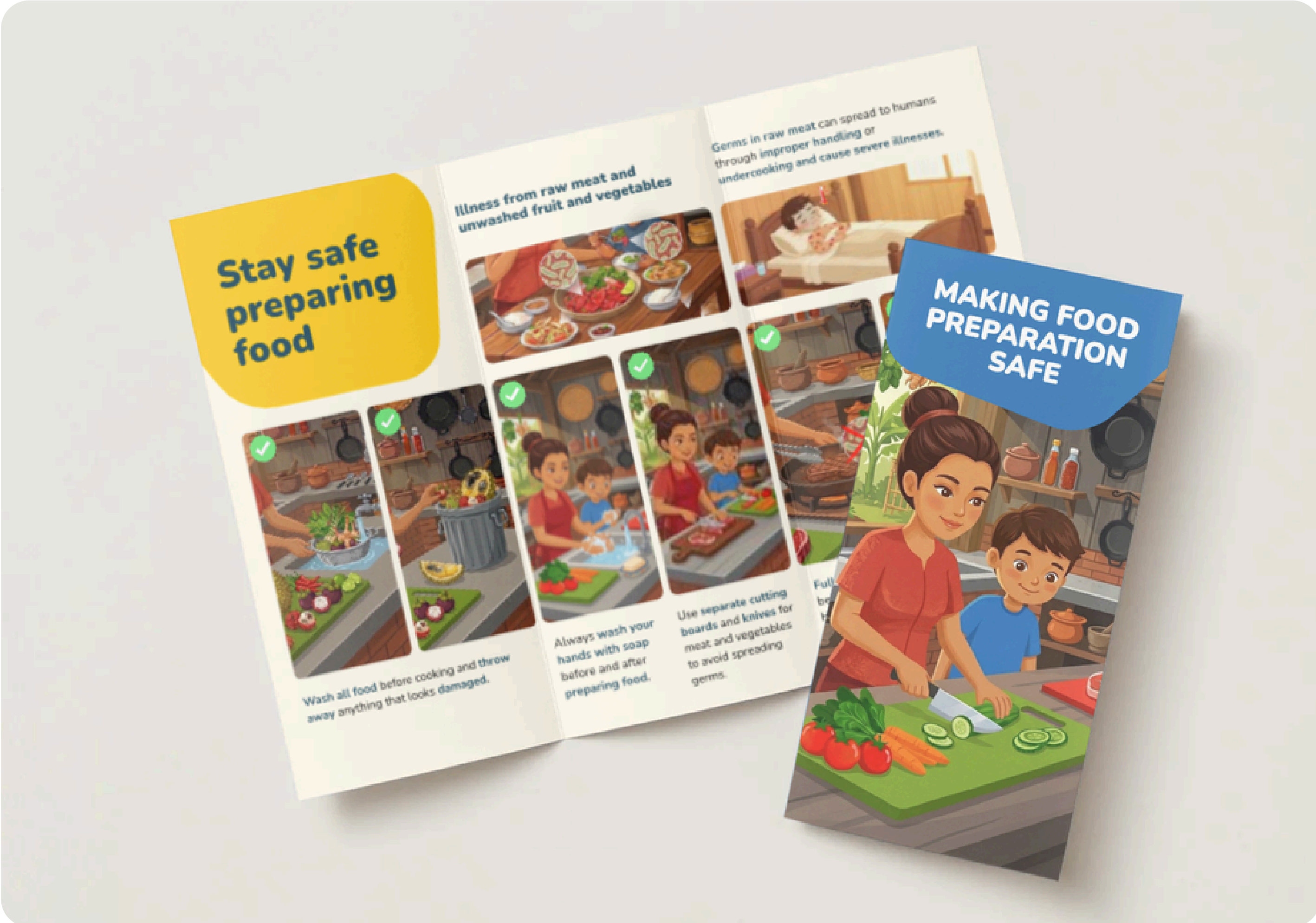
Stay safe preparing food
Illness from raw meat and unwashed fruit and vegetables
Germs in raw meat can spread to humans through improper handling or undercooking and cause severe illnesses.
Wash all food before cooking and throw away anything that looks damaged.
Always wash your hands with soap before and after preparing food.
Use separate cutting boards and knives for meat and vegetables to avoid spreading germs.
MAKING FOOD PREPARATION SAFE

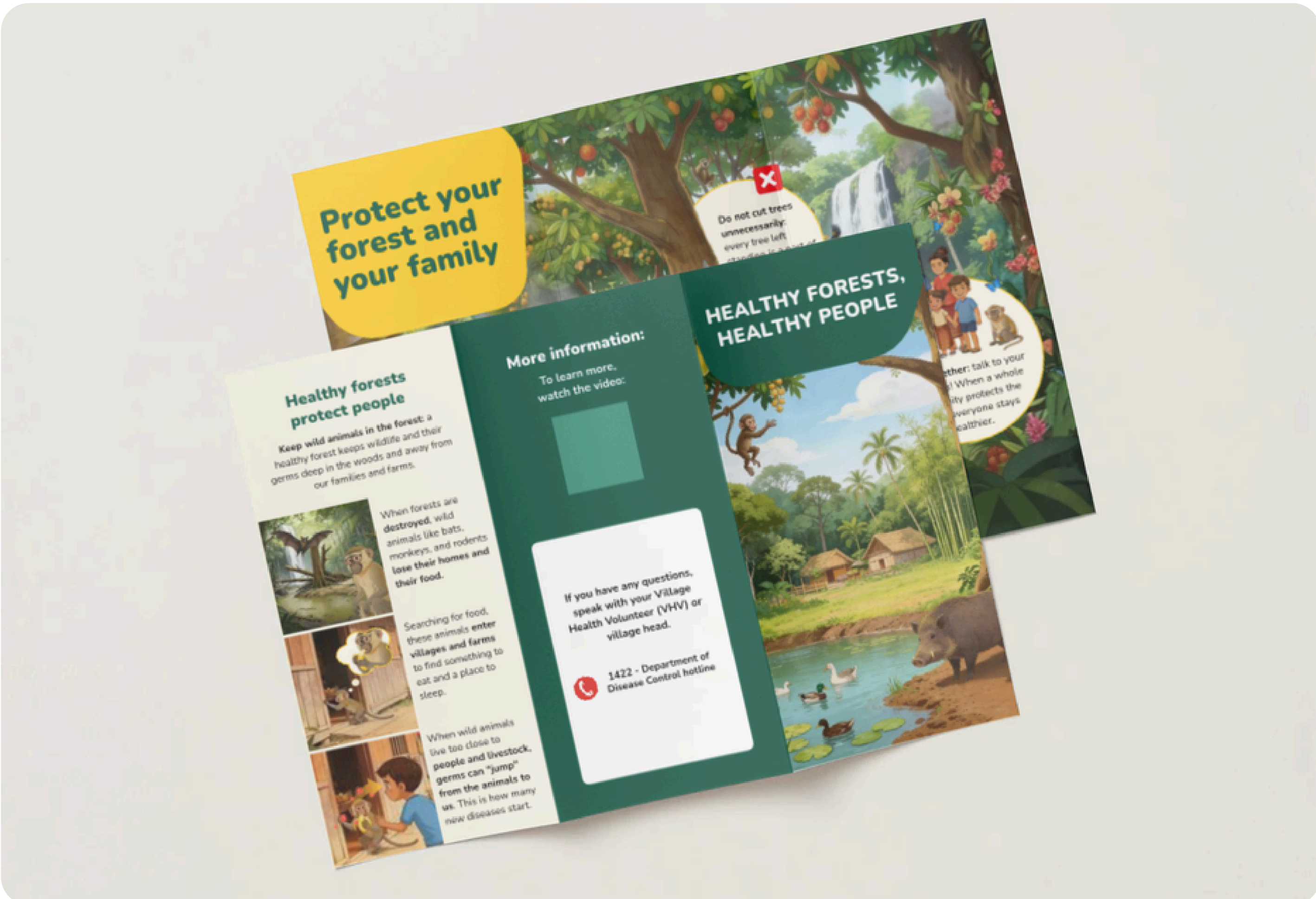
Protect your forest and your family
Do not cut trees unnecessarily:
HEALTHY FORESTS, HEALTHY PEOPLE
Healthy forests protect people
Keep wild animals in the forest: a healthy forest keeps wildlife and their germs deep in the woods and away from our families and farms.
When forests are destroyed, wild animals like bats, monkeys, and rodents lose their homes and their food.
Searching for food, these animals enter villages and farms to find something to eat and a place to sleep.
When wild animals live too close to people and livestock, germs can "jump" from the animals to us. This is how many new diseases start.
More information:
To learn more, watch the video:
If you have any questions, speak with your Village Health Volunteer (VHV) or village head.
1422 - Department of Disease Control hotline
