# Supplementary material for: Community-tailored One Health educational intervention to enhance knowledge and practices for zoonotic disease prevention in rural Thailand: A protocol for a prospective cluster randomised controlled Trial in Chanthaburi, Thailand (Saan Suk trial)
Source: PLoS One. 2026 Sep 15;21(9):e0354989. doi: 10.1371/journal.pone.0354989 (PMC13577416; doi:10.1371/journal.pone.0354989)
Supplement: Supplementary file 3 [file pone.0354989.s003.docx]

Inclusivity in global research

PLOS’ policy on inclusivity in global research aims to improve transparency in the reporting of research performed outside of researchers’ own country or community and ensures that PLOS publications reporting global research adhere to high standards for research ethics and authorship. Authors of relevant research articles may be asked to complete the questionnaire below, which outlines ethical, cultural, and scientific considerations specific to inclusivity in global research. This questionnaire may be requested when researchers have travelled to a different country to conduct research, if research uses samples collected in another country, research with Indigenous populations or their lands, or if research is on cultural artefacts. Researchers travelling to another country solely to use laboratory equipment will not normally be required to complete the questionnaire. However, the questionnaire can be requested at the journal’s discretion for any submission – if you have been requested to complete this questionnaire by the PLOS journal you submitted to, please do so.

Please complete the questionnaire below and include this as a Supporting Information file with your manuscript. Note that if your paper is accepted for publication, this checklist will be published with your article in the supporting information files. Please ensure that you reference the checklist in the main body of your manuscript. We suggest adding a subsection ‘Inclusivity in global research’ to your Methods section and adding the following sentence: “Additional information regarding the ethical, cultural, and scientific considerations specific to inclusivity in global research is included in the Supporting Information (SX Checklist)”

The questions have been designed to be applicable to a wide range of study types, and there are subsections for both human subjects research and non-human subjects research. If any of the questions are not relevant to your research please mark them as “N/A” as appropriate.

**Ethical considerations, permits and authorship**

*This section is applicable to all research types.*

Provide details as to who granted permissions and/or consent for the study to take place in the Methods section of your manuscript. This should include the names of **all** ethics boards, governmental organizations, community leaders or other bodies that provided approval for the study. If individuals provided approval refer to these people by their role or title but do not list their name(s).

Reported on page numbers: 10, 19, 23.

The study receives governmental support from Provincial and District-level administration. Community leaders from the 24 villages involved in the study, as well as Village Health Volunteers (VHVs), household heads, and individuals participating in the study, will sign informed consent forms before taking part in any activity. Ethical approval was granted by the Ethics Commission of the Medical Faculty of Heidelberg University, Germany (reference S-607/2025; approval dated 14 April 2026).

If there were any deviations from the study protocol after approval was obtained please provide details of these changes in the Methods section of your manuscript.
Did this study involve local collaborators that are residents of the country where the research was conducted or members of the community studied? If you do not have any authors from said communities, please provide an explanation for this below.

Study authors include six researchers who are Thai and/or based in Thailand, at Khon Kaen University and Kranuan Crown Prince Hospital.

Reported on page number: N/A. At the time of submission, no deviations had occurred from the study protocol after approval was obtained.

Everyone listed as an author should meet PLOS’ criteria for authorship and all individuals who meet these criteria should be included in the author byline, rather than the acknowledgements. For further information please see the journal’s Authorship Policy.

**Human subjects research (e.g. health research, medical research, cross-cultural psychology)**

Did you obtain written informed consent from a representative of the local community or region before the research took place? How did you establish who speaks for the community? Details of written informed consent obtained from study participants should be reported separately in the Methods section of your manuscript.

Written informed consent will be obtained from each Village Head before village (cluster) enrolment and randomisation. Village Heads are the formally recognised leaders within Thailand's local administrative structure and are therefore appropriate community representatives. VHV Heads also sign a written consent form representing the Village Health Volunteer network at the cluster level. Details are reported in pages 9-12 in the Methods section of the manuscript.

How did members of the local community provide input on the aims of the research investigation, its methodology, and its anticipated outcome(s)?

When engaging with the local community, how did you ensure that the informed consent documents and other materials could be understood by local stakeholders?

Participant-facing documents, such as informed consents and intervention materials (leaflets and videos) are translated by certified language translators and provided to the community in Thai language. Trained Thai-speaking researchers explain the study, present the materials, answer questions, and confirm understanding before consent.

The Saan Suk intervention was co-developed with the local communities through the Human-Centered Design (HCD) approach (see pages 12-13). Before the intervention was designed, qualitative and quantitative data were collected to understand the local context and identify knowledge and behavioural gaps. Community members then contributed through an ideation workshop and a pilot implementation. Their input and feedback directly informed the intervention content and materials, the delivery format, culturally appropriate content, and considerations related to feasibility and implementation in the field.

Will the findings of the research be made available in an understandable format to stakeholders in the community where the study was conducted (e.g. via a presentation, summary report, copies of publications, etc.)? Please provide details of how this will be achieved.

Findings will be shared with participating villages, VHVs, and local and national health authorities through community feedback sessions and a Thai-language summary. Results will also be available through the project website, open-access publications, presentations at local conferences, and meetings with local stakeholders as part of the PANDASIA stakeholder engagement activities. See page 21.

**Non-human subjects research using specimens/ animals collected as part of the study, or those housed in archival collections. Examples include archaeology, paleontology, botany and zoology.**

Did the permission you obtained from a local authority to perform the study include an agreement on access to outputs and benefit sharing? This may include procedures to enable fair distribution of the benefits and resources arising from the research performed. Please include any details of Prior Informed Consent and Benefit Sharing Agreements obtained. These may be required by field-specific regulations, for example the Convention on Biological Diversity (CBD) and the associated Nagoya Protocol.

N/A.

If the material used in your study was imported, please A) provide the year it was imported and B) indicate whether permits were obtained to import/export the materials used, C) provide details of any permits obtained. If this information is not available, please indicate this.

N/A.

If you used archival specimens, please state how the material used in your study was acquired by the institute it is held in and provide details of any permits obtained for the original excavations/ sample collection. If this information is not available, please indicate this.

N/A.

How was the potential cultural significance of the materials collected in your study to local communities considered in your research design? Were Indigenous peoples and/or local researchers and institutions involved with archaeological excavations / collection of specimens? If so, please provide a description of their involvement.

N/A.

If your manuscript includes photographs of human remains please indicate whether authors obtained permission from descendants or affiliated cultural communities to do so.

N/A.
